# Aesthetic Submandibular Gland Resection: A Review of Complication Incidence and Prevention

**DOI:** 10.1093/asj/sjaf096

**Published:** 2025-05-27

**Authors:** Sara B A Morel, Luis H Macias, Ritu Chopra, Joshua Vorstenbosch, Tyler Safran

## Abstract

Achieving optimal neck contour during facial rejuvenation may require addressing the submandibular glands, as supraplatysmal fat removal alone is insufficient for many patients, particularly those with fuller necks. The purpose of this study is to review the rate of complications associated with removal and techniques to improve safety. A comprehensive search was conducted to identify literature on complications associated with submandibular gland removal in the context of facial rejuvenation. Studies were included if they provided patient data and complication rates following surgical outcomes. Screening of 908 articles identified 11 (1.21%) studies on complication rates related to submandibular gland resection for aesthetic purposes. A total of 3379 participants were included, of which 48.86% (*n* = 1651) underwent submandibular gland resection, with a mean age of 50.4 years (range, 32-83), and 88.53% (*n* = 247) were female. Complications included hematomas (1.15%, *n* = 16), requiring reoperation in 23.08% (*n* = 3) of cases, sialoceles (1.33%, *n* = 21), salivary fistulas (0.82%, *n* = 6), nerve injury (3.97%, *n* = 40), xerostomia (0.13%, *n* = 1), and neck induration (21.43%, *n* = 21), with complication rates varying across studies. Submandibular gland reduction offers aesthetic benefits but comes with potential risks. Effective preoperative planning, meticulous gland mobilization, and proper exposure are essential for minimizing risks. Advances in techniques, such as improved dissection methods, the use of botulinum toxin, netting techniques, and energy-based instruments like LigaSure (Medtronic, Minneapolis, MN), have enhanced the safety of the procedure. While complications can arise, they typically resolve within a few months, and the overall outcomes improve with surgical experience. The associated risks and benefit profile should be discussed thoroughly with patients.

**Level of Evidence: 3** (Therapeutic)

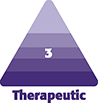

Achieving an optimal neck contour during facial rejuvenation often requires addressing the submandibular glands. It has been shown that supraplatysmal fat removal alone is insufficient for many patients, particularly in patients with fuller necks. In these cases, failure to address deeper structures like subplatysmal fat, the digastric muscle, and submandibular glands can result in unsatisfactory outcomes.^1^ The submandibular gland resection has long been feared given its proximity to facial nerves and vessels, as well as sizable calibre vessels that need to be controlled at time of resection.

Usually, submandibular glands, located beneath the mandibular border, do not require intervention. However, when they become ptotic or enlarged, they can significantly contribute to neck fullness and hinder the desired contour. This is especially relevant for patients for whom glandular prominence may become more noticeable after other aspects of the neck are addressed.^2,3^ Interestingly, submandibular gland surgery is twice as frequent in secondary or tertiary facelift patients, suggesting inadequate attention to the gland in primary procedures or resection of surrounding deep structures without addressing the gland itself.^3^

The removal of the submandibular gland for aesthetic purposes was first described by Connell in 1987 as a technique to enhance neck contour and achieve a more youthful appearance.^4^ Since then, the practice has sparked debate among surgeons. While some argue that excising the submandibular gland is crucial in certain cases to improve neck aesthetics, many contend that this approach is not always necessary.^5^ A survey of Aesthetic Society members found that 89% of 60 facelift surgeons avoid gland excision primarily due to concerns about danger to the patient, with 78% deeming it borderline to always unacceptable.^6^ Surgeons who advocated not to perform resection perceived complication rates between 0.01% and 30%, with marked peaks at 1%, 5%, and 10%.^7^

Various methods have been proposed to address this issue over the years, including attempts to reposition the gland through tacking or releasing it from its attachments, though these techniques have often been proven unreliable.^8^ Since the glands sit between the stronger mylohyoid muscle and the weaker platysma muscle, it cannot be relocated under the mandibular border.^9^

The goal of this article is to present the latest data on partial aesthetic submandibular gland resection both to facilitate the informed consent with the patient as well as guide the surgeon on safe removal.

## METHODS

This review involved a search of the literature in the PubMed (US National Library of Medicine, Bethesda, MD) database,^10^ covering studies from database inception up until November 15, 2024. The search strategy included the following combinations of terms: (“anatomy” AND “submandibular gland”), (“anatomy” AND “submandibular gland” AND (“face lift” OR “neck lift”)), and ((((((facelift) OR (neck lift)) OR (face lift)) OR (neck lift)) OR (facial rejuvenation)) AND ((submandibular gland) OR (gland))). Studies were eligible for inclusion if they specifically addressed complication rates associated with the removal of the submandibular gland for aesthetic purposes. The initial screening of titles and abstracts was conducted independently by 2 reviewers (S.B.A.M. and T.S.), with any discrepancies resolved through discussion. Only full texts that describe patient data and complication rates were included for analysis. The references of all included articles were also screened for potential inclusion. Data extraction was carried out using a standardized Microsoft Excel spreadsheet (Microsoft Corporation, Redmond, WA).^11^ The initial extraction was performed by 1 reviewer (S.B.A.M.), and the data were independently verified for accuracy and completeness by a second reviewer (T.S.). Statistics were performed on the overall compiled patient data. All data were extracted into an Excel document, where the total number of patients, glands, and patients per gender were recorded, as reported in the individual studies. The mean age was recorded for all studies that provided this information, with a weighted mean age calculated across all participants. Complication rates were calculated as weighted means, with weights corresponding to the number of participants undergoing gland resection in each study that reported said complications. This systematic review was conducted according to the Preferred Reporting Items for Systematic Reviews and Meta-Analysis (PRISMA) guidelines.^12^ The PROSPERO registration number was CRD420251043098.

## RESULTS

Screening of the literature revealed 908 articles, of which 11 (1.21%) studies looking at complication rates in relation to submandibular resection for aesthetic purposes were included (Supplemental Figure 1). There were 10 retrospective studies (90.91%), and 1 randomized control trial (9.09%). Findings from each study as per surgical technique and complications are noted in Supplemental Table 1.

### Demographics

Across all studies, there was a total of 3379 participants, of which 48.86% (*n* = 1651) underwent submandibular gland resection. In the cohort of patients who underwent submandibular gland resection, the mean age was 50.4 years old (range, 32-83). Out of the studies who declared gender for the cohort undergoing submandibular gland resection (*n* = 279), 88.53% (*n* = 247) were female, and 11.47% (*n* = 32) were male.

### Surgical Technique

All cases were performed through a submental approach. Out of the studies, 45.45% (*n* = 5) placed the incision in the submental crease,^1,3,5,8,13^ whereas 54.55% (*n* = 6) placed it behind the crease.^14-19^ Regarding the devices used, 27.27% (*n* = 3) employed a monopolar device,^1,13,14^ 18.18% (*n* = 2) used a bipolar device,^5,15^ 18.18% (*n* = 2) did not specify the device,^13,19^ 9.09% (*n* = 1) used both monopolar and bipolar devices,^7^ 9.09% (*n* = 1) used a device with both monopolar and bipolar properties (Valleylab Force FX, Medtronic, Minneapolis, MN),^17,20^ 9.09% (*n* = 1) used a LigaSure (Medtronic),^5,21^ and 9.09% (*n* = 1) used a LigaClip (Ethicon, Raritan, NJ).^16^ ^22^

### Complications

The complication rates were analyzed and categorized based on the surgical techniques used (Table 1). Out of all patients who underwent submandibular gland resection, hematomas occurred in 1.15% (*n* = 16) of cases (range, 0.00%-8.00%). Out of these, surgical evacuation and reoperation occurred in 23.08% of cases (*n* = 3) and 1 of these cases (*n* = 1) was considered life-threatening.^3^ Sialoceles occurred in 1.33% (*n* = 21) of the cases (range, 0.00%-9.52%), salivary fistulas occurred in 0.82% (*n* = 6) of the cases (range, 0.00%-4.46%) across studies, transient nerve injury occurred in 3.97% (*n* = 40) of the cases (range, 0.00% and 8.43%) across studies, and xerostomia occurred in 0.13% (*n* = 1) of the cases (range, 0.00%-0.89%) across studies with all cases resolving within 2^16^ to 15 weeks.^9^ Lastly, postoperative induration of the neck occurred in 1.27% (*n* = 21) of overall cases (range, 6.25%-7.23%).^1,5^

**Table 1.** Complication Rates

Complication	Number of cases	Incidence (%)	Range (%)
Hematoma	16	1.15	0.00-8.00
Sialocele	21	1.33	0.00-9.52
Salivary fistula	6	0.82	0.00-4.46
Mandibular nerve injury	40	3.97	0.00-8.43
Xerostomia	1	0.13	0.00-0.89
Induration of the neck	21	21.43	6.25-7.23

## DISCUSSION

This study highlights the latest data describing the aesthetic removal of the submandibular glands. The main complications identified by the authors include haematoma, sialocele, salivary fistulas, transient nerve injury, xerostomia, and induration of the neck. Multiple advances in technique and anatomical understanding may help the surgeon remove these glands in a safe and predictable fashion.

### Surgical Anatomy of the Submandibular Gland

**Figure 1.**
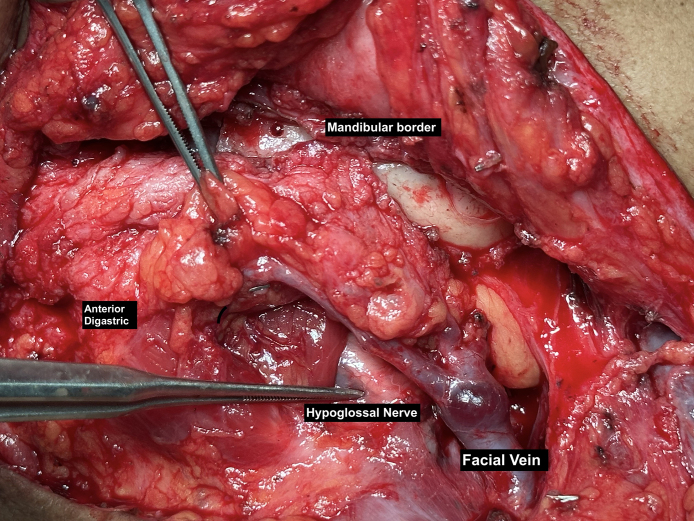
View of submandibular gland via submental incision and subplatysmal dissection (52F).

Anatomic understanding of the submandibular gland is of paramount importance to safely perform this procedure. The submandibular gland is located within the submandibular triangle, a region bordered by the anterior and posterior bellies of the digastric muscle and the inferior border of the mandible.^23,24^ This triangular space contains the gland, lying between the digastric muscles, above the mylohyoid muscle, and deep to the platysma and superficial cervical deep fascia (Figure 1).

The blood supply to the submandibular gland primarily comes from the submandibular artery off the facial, which enters the gland superolaterally.^25^ During conservative resection, only the terminal branches of this artery extending into the gland are typically encountered.^18^ A central perforator artery, from either the facial or lingual artery, penetrates the posterior deep lobe and enters the superficial lobe.^26^ Careful dissection is crucial around these vessels, which must be isolated and controlled.^25^ Additional vascular structures include the facial artery and vein, located on the deeper and lateral aspects of the gland's capsule.

In terms of aesthetic resection, the major nerve to be protected is the marginal mandibular nerve. The marginal mandibular nerve crosses superficial to the facial vein on the posterior surface of the platysma, situated ∼3.7 cm above the inferior limit of the submandibular gland.^25^ Much variation occurs in the nerve. In a study of 50 cadavers, the marginal mandibular branch of the facial nerve had a single branch at the origin in 88% of cases, but 84% of cadavers had more than 2 branches at termination.^27^ It was always superficial to the facial artery and vein. The nerve was found above the inferior border of the mandible in 81% of the cases when anterior to the facial artery, with a distance from the mandible between 0.9 and 1.4 cm. Anastomoses with the buccal branch of the facial nerve occurred in 12% of cases and with the mental nerve in 28% of cases.^27^

### Complications With Submandibular Gland Resection

#### Haematoma

Haematomas are a significant complication of submandibular gland resection and occurred in 1.15% (*n* = 16) of cases. Interestingly, a survey of plastic surgeons performing facelifts found that hematomas were believed to be the most common complication.^7^ This complication is due to challenges in controlling bleeding, especially given the calibre of vessel and anatomical location. Gland resection requires meticulous dissection in the posterolateral aspect of the gland to minimize the risk of bleeding, with special attention from the anaesthesiologist to control blood pressure during this phase of the procedure.

If uncontrollable arterial bleeding occurs, the surgeon should apply a cold saline gauze and pressure for 5 min. The bleeding should slow to the point of being amenable to visualization for cauterization.^5^ Venous bleeding is managed by placing the patient in a reverse Trendelenburg position to reduce venous pressure before cauterizing the vein.^14^ Local anaesthetic containing epinephrine should be preinjected into the gland to reduce the response to traction on the gland and bleeding.^3,28,29^ A second suction setup with a Yankauer suction should be available for use during resection if bleeding occurs as well as excellent visualization with the help of trained assistants.^25^

To reduce the risk of hematomas, a transcutaneous traction suture technique at the caudal aspect of the submandibular gland to control bleeding and maintain access was proposed.^29,30^ A quilting suture technique that seals the platysma against the remaining submandibular gland and mylohyoid muscle, eliminating any dead space and therefore preventing hematoma formation was introduced.^18^ This technique was demonstrated, with no major hematomas reported in 523 patients who underwent submandibular gland resection. Deep hematomas may also have airway reprocutions.^13^ Preoperative management, including cessation of medications and hypertension control is paramount. Two cases requiring early urgent reoperation of which 1 was life-threatening were described.^3^ Special attention was given to the central intraglandular artery, with ligature preferred over cauterization for better haemostasis. Suction drainage was found to be ineffective for major arterial bleeding.^3^

Hematomas may occur up to 10 to 14 days postoperatively, although they are rare and typically do not present with overlying bruising since they are found in the subplatysmal space.^31^ Postoperatively, drains (both below and above platysma) are suggested to be placed for at least 24 h to prevent seroma formation. Microfoam tape (3M, St Paul, MN) has also been suggested by 1 author to be applied for 1 week to help reduce submental swelling.^8^ Minor cervical hematomas may also be treated with drainage and netting sutures.^15^

#### Sialocele

Sialoceles are a known complication in partial resection of submandibular glands during neck lift surgery and occurred in 1.33% (*n* = 21) of cases.

Conservative submandibular gland resection is the key to minimize sialoma risk.^1^ All cases were managed conservatively with pressure dressings, needle aspiration, and botulinum toxin injections.^18^ One group attributes the low incidence of sialocele to their technique, which involves placing continuous haemostatic sutures and closing the gland capsule to encourage proper drainage and prevent leakage.^18^ Another case was treated with a percutaneous Angiocath drain for 1 week.^8^ One author injected 5 to 10 units of botulinum toxin into the raw surface of each gland post-resection, which reduced the incidence of both sialoma and salivary fistula to 2.4% of cases compared with 24% of patients in a control group from the same author.^13,17^ The authors also describe suturing the platysma muscle over the remaining gland and mylohyoid muscle, effectively sealing the surgical area and preventing saliva accumulation.^17^ This quilting suture technique is also applied to promote proper drainage of gland secretions into the mouth, utilizing a natural pressure gradient.^15^ Another study used 8 units of botulinum toxin, and reconstructing the anterior capsule of the remaining submandibular gland was effective in preventing sialocele formation.^16^

Overall, these accumulations of saliva are managed with serial percutaneous aspiration compressive dressings and the application of botulinum toxin, either prophylactically or postoperatively, to inhibit salivary secretion.^14,17,23^ Management may also include dietary restrictions and trial of scopolamine patches.^15^ Additionally, a temporary haemostatic net can be used to occlude dead space during the resolution of the sialocele, and a salivary resting diet may also help reduce incidence.^32^

#### Nerve Injury and Paralysis

Mandibular nerve injury occurred in 3.97% (*n* = 40) of cases. Temporary marginal mandibular nerve paresis typically affects the lower lip depressor muscle, may result from other face-lift manoeuvers such as the release of the mandibular retaining ligament, which is closely associated with the marginal mandibular branch of the facial nerve.^32^ Transient denervation of the platysma muscle may also contribute to similar findings, though this usually resolves spontaneously.^32^

It has been argued that since the marginal mandibular nerve is at the cranial border of the capsule, any twitching seen during resection is likely due to stimulation of the cervical nerve, which runs through the deep cervical fascia into the surrounding platysma, or platysma muscle itself.^33^ This can occur from the use of monopolar cautery, heat dissipation, or from retractor traction.

One possible solution is the use of bipolar cautery or Ligasure to avoid thermal spread and injury. Additionally, the region should be intermittently irrigated with cold saline to cool the surrounding tissues after resection.^15^ Neurapraxia was reported to have a higher incidence in secondary neck-lift patients (10%) compared with primary neck-lift patients (2.5%).^3^ The condition resolved completely within 3 months, and botulinum toxin was used in 3 patients to alleviate contralateral lip hyperactivity.

#### Xerostomia

Xerostomia occurred in 0.13% (*n* = 1) of cases as observed in Mendelson's study.^3^ As dry mouth is a possible complication of submandibular gland resection, for patients undergoing neck lift with gland reduction, a salivary rest diet is advised, consisting of soft, moist foods that are easy to swallow. Patients should avoid stimulating foods, such as sweet, salty, sour, dry, or citrus for 7 to 10 days to prevent fluid collections and neck induration.^25^ Alcohol consumption should be avoided for at least 2 weeks post-surgery.^25^

#### Prolonged Swelling and Induration

Induration of the neck occurred in 21.43% (*n* = 21) of cases. Prolonged induration may be the result of disrupted lymphatics, inflammation from excessive cautery, residual hematoma, or seroma. In some cases, induration can take up to 3 months to resolve, which was demonstrated in a study where 15 out of 25 patients had prolonged swelling and 6 required aspiration.^17^ Haemostatic netting is hypothesized to help reduce postoperative oedema.^5,8^ For more resistant swelling, corticosteroid injections like Kenalog or 5-Fluorouracil, diluted with lidocaine for pain relief, can hasten recovery, with repeated injections every 2 to 3 weeks as needed.^5,34^ LigaSure may help with induration by sealing lymphatics as subplatysmal tissues are excised.^33^ Additionally, postoperative massage and careful attention to positioning and head movement can improve neck flexibility and appearance, particularly when a submental incision is used.^32^

#### Prevention

**Figure 2.**
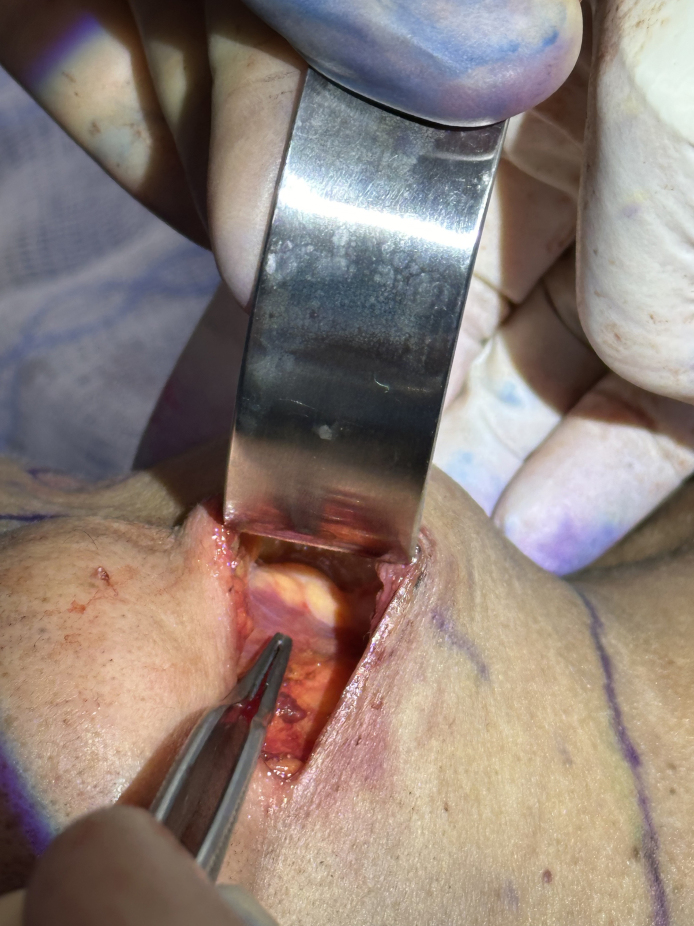
In vivo dissection of submandibular space status-post submandibular gland removal (56F).

The best strategy to reduce complication in submandibular gland resection is by optimizing operative set-up. A submental incision was used to access the subplatysmal space from medial to lateral until the lateral extent of the gland. Visualization needs to be maximized by leveraging 1-inch malleable ribbon retractors, insulated instruments, and trained assistants.^26^ In some cases, the authors recommend suturing the medial platysma to the skin temporarily to help with retraction^15^ (Figure 2).

The gland capsule should be infiltrated with 1% lidocaine with epinephrine and given ample time to allow for vasoconstriction. The dissection proceeds within the gland capsule and the superficial lobe is mobilized inferiorly, with careful dissection of 1 to 3 vessels entering the medial aspect of the lobe and a central perforator from the deep lobe. These vessels are dissected and cauterized before resection either with monopolar, bipolar or Ligasure. Vascular clips should be on standby. The superficial lobe is partially resected, and the base of the remaining gland is cauterized to ensure haemostasis and minimize the risk of salivary fistula. In some cases, the submandibular capsule has been described to be closed in a variety of techniques, or the platysma is sutured to the underlying structures.^26^

One study assessed the use of LigaSure, a bipolar energy-based instrument, for submandibular gland excisions.^5^ The authors found that the use of this instrument reduced thermal injury and surgical time, significantly decreasing risks of hematoma and glandular inflammation. LigaSure is highly effective because it provides reliable vessel sealing with minimal thermal spread, limiting heat diffusion to a 2 mm zone, can coagulate vessels up to 7 mm and withstanding pressures up to 3 times systolic blood pressure, which significantly reduces tissue damage and enhances bleeding control. This energy-based system offers a substantial improvement over previous methods, such as monopolar cautery, which often resulted in inadequate haemostasis, tissue charring, and increased glandular fragility, leading to higher bleeding risks in 15% to 20% of cases. While bipolar cautery also improved control, it still required multiple applications, which caused inflammation, prolonged operative times, and compromised tissue integrity. In contrast, LigaSure typically requires only 2 to 3 applications, eliminating the need for additional ligation and ensuring no perioperative bleeding or hematomas, except for 2 localized swellings observed in the study. Additionally, the average excision time was of 3.8 min per gland. Temporary lower lip weakness occurred in 7 patients but 1 recovered fully within 3 months, and the remaining case recovered within 6 months. Furthermore, compared with monopolar cautery, LigaSure led to fewer cases of seroma and induration, likely due to its reduced thermal effect and ability to seal gland ducts effectively. One note is that the jaws are not wide enough to encompass the entire gland, leading to peripheral bleeding. To address this, the authors recommend using monopolar cautery to score the anterior and posterior gland, narrowing it and controlling peripheral bleeding, while allowing the device to seal central perforating arteries (Video).

### Adjunctive Use of Botulinum Toxin

As mentioned above, the use of botulinum toxin led to a marked reduction in sialocele rates post-surgery compared with higher rates seen in other studies.^16^ Botox (Allergan, Inc., an AbbVie company, Irvine, CA)^35^ has emerged as a valuable adjunct therapy following submandibular gland resection. Botox inhibits acetylcholine release at the neuroglandular junction, reducing salivary secretion.^36^

As a less invasive strategy, Botox was evaluated as an adjunctive treatment for benign submandibular gland hypertrophy.^6^ The study included 23 patients with sialadenosis, sialolithiasis, or aesthetic concerns regarding the prominence of their submandibular gland. Botox injections, typically 15 units per gland, were administered under ultrasound guidance. The results were highly favourable, with reductions in gland size ranging from 0.3 to 1.2 cm observed within 2 to 10 days after treatment. The most substantial reduction was seen in the height of the glands, contributing to a more refined neck contour and improved jawline definition.^6^ Importantly, the treatment did not cause significant complications. Minor side effects included temporary tension, hypersensitivity, bruising, and mild pain during the injection, but there were no reports of more severe adverse effect.^6^ The effects of Botox were not only rapid but also long-lasting, with many patients reporting sustained improvements without the need for additional injections.

### Limitations

One major limitation to this study is that many of the studies are written and performed by key opinion leaders in the field. Many surgeons will argue that the true rate of complications when removing glands may in fact be higher than described above, especially given the steep learning curve. Surgeons are encouraged to seek training and engaged in proper discussion with their prospective patient prior to performing gland removal.

## CONCLUSIONS

The decision to perform submandibular gland reduction should be made collaboratively with the patient after a thorough discussion of its risks and benefits.^25^ Patients should be informed of potential complications, including oedema, bleeding, sialoma, salivary fistula, and dry mouth, though these are rare. It should also be emphasized that only the protruding portion of the gland is excised, leaving most of the gland intact.^25^ Effective preoperative planning, gland mobilization, and proper exposure are essential for controlling bleeding. Although complications like neurapraxia, sialocele, and dry mouth can occur, they are typically temporary and resolve within a few months. With experience, complication rates decrease, and the procedure becomes more effective and predictable.^3^

## Supplemental Material

This article contains supplemental material located online at https://doi.org/10.1093/asj/sjaf096.

## Supplementary Material

sjaf096_Supplementary_Data
