# Supplementary material for: Aesthetic Submandibular Gland Resection: A Review of Complication Incidence and Prevention
Source: Aesthet Surg J. 2025 May 27;45(9):869–74. doi: 10.1093/asj/sjaf096 (PMC12451699; doi:10.1093/asj/sjaf096)
Supplement: sjaf096_Supplementary_Data [file sjaf096_Supplementary_Data.zip › Aesthetic Submandibular Gland Resection_PRISMA.docx]

**Supplemental Figure 1: PRISMA SEARCH STRATEGY**

**Identification of studies via databases and registers**

Records removed *before screening*:

Duplicate records removed (n = 0)

Records marked as ineligible by automation tools (n = 0)

Records removed for other reasons (n = 0)

Records identified from: PubMed

Databases (n = 1,332)

**Identification**

Records screened

(n = 1,332)

References screened (n =20)

Records excluded

(n = 1341)

Reports sought for retrieval

(n = 11)

Reports not retrieved

(n = 0)

**Screening**

Reports assessed for eligibility

(n = 11)

Reports excluded: 0

Studies included in review

(n = 11)

**Included**
