# Supplementary material for: Aesthetic Submandibular Gland Resection: A Review of Complication Incidence and Prevention
Source: Aesthet Surg J. 2025 May 27;45(9):869–74. doi: 10.1093/asj/sjaf096 (PMC12451699; doi:10.1093/asj/sjaf096)
Supplement: sjaf096_Supplementary_Data [file sjaf096_Supplementary_Data.zip › Aesthetic Submandibular Gland Resection_SupplementaryTable1.docx]

**Table 1: Literature Review Including Resection Method, Complication Rates and Notes on Outcomes**

| **Author** | **Reference** | **Year of Publication** | **Type of Study** | **Study Demographics** | **Resection Technique** | **Hematoma** | **Sialocele** | **Salivary fistula** | **Mandibular Nerve Injury** | **Xerostomia** | **Induration** | **Comments** |
| --- | --- | --- | --- | --- | --- | --- | --- | --- | --- | --- | --- | --- |
| **De Pina** | **15** | **1991** | **Retrospective** | Participants: 12 with SMG resection  Female: 8  Male: 4  Age: 35-65  Approach: Submental  Incision: 4cm below the lower edge of mandible, Submental crease  Monopolar, Bipolar, Ligature: NR | The enlargement or ptosis of the submandibular gland is managed well with anterior and posterior plication of the platysma muscle. However, when plication is insufficient, a partial resection may be required. A 3.5 cm horizontal skin incision is made 4 cm below the lower edge of the mandible, either through a direct approach or in association with a rhytidoplasty procedure. The platysma muscle is dissected, and the gland is carefully exposed, ensuring preservation of the mandibular branch of the facial nerve and the hypoglossal nerve. The gland is enucleated after dissection, with the remaining sutured in place. | 0 | 0 | 0 | 0 | 0 |  |  |
| **Bravo** | **16** | **2013** | **Retrospective** | Participants: 21 with SMG resection out of 27 facelifts  Female: 21 (77.8%)  Male: 6 (22.2%)  Age: 34-81  Approach: Submental  Incision: Submental crease  Monopolar, Bipolar, Ligature: NS | Submandibular gland reduction was performed through a submental approach, ensuring adequate release of its capsule. For partial parotid gland resection, the procedure was done through a periauricular facelift approach, ensuring that the facial nerve remained unaffected. | 0 | 2 (9.52%) |  | 1 (4.76%): Weakness of depressor anguli oris | 0 |  | Transcutaneous drainage for sialoceles  Treatment for salivary fistula  Observation of spontaneous resolution for facial nerve weakness |
| **Feldman** | **7** | **2014** | **Retrospective** | Participants: 129 with SMG resection out of 522 necklifts  Female: 449 (86%)  Male: 73 (14%)  Age: NR  Approach: Submental: 486 (93%)  Incision: Submental crease  Monopolar, Bipolar | The resection involves carefully maintaining the integrity of the deep lobes and part of the superficial lobes to minimize complications (129 cases, 24.7%). Corset Platysmaplasty was performed in 470 of cases (90%). | No deep hematomas causing postoperative airway problems | Percutaneous drain: 1 (0.78%) |  |  | 0 |  | No intraoperative emergencies or deep hematomas causing postoperative airway issues were observed  The procedure also ensured that no patients complained of dry mouth post-surgery. |
| **Mendelson** | **3** | **2015** | **Retrospective** | Participants: 112 with SMG resection out of 736 necklifts  Female: 97 (87%)  Male: 15 (13%)  Age: 57 (38-80)  Approach: Submental  Incision: Submental crease  Ligature | The technique involved elevating the skin flap off the platysma surface, defining the medial platysma edges, and continuing dissection on the undersurface of the platysma to mobilize the muscle for lateral retraction. Excess fat is removed, and the anterior belly of the digastric muscle is defined. The submandibular gland is accessed by incising the deep fascia at the lateral border of the digastric muscle. The gland was mobilized from its capsule using blunt-tip scissors, diathermy, and atraumatic clamps, with countertraction from a retractor. Local anesthesia with epinephrine was used to reduce bleeding. The parenchymal reduction was carried out using a diathermy blade, with the central artery of the gland ligated to ensure hemostasis. A suction drain was used initially, but later only for specific indications, with partial closure of the residual cavity. |  | 5 (4.46%) | 5 (4.46%) |  | 1(0.89%): Persistent for almost 6 months |  | 2 patients were reoperated (life-threatening in 1 patient) |
| **Auersvald** | **13** | **2015** | **Randomized Control Trial** | Participants: 240 with SMG resection  Control: 25  Study Group: 215  Female: NR  Male: NR  Age: NR  Approach: NR  Incision: Submental, 2-3 cm behind the submental crease  Monopolar and Bipolar: Force FX, Valleylab, Mass | A new surgical technique for partial resection of the superficial lobe of the submandibular gland involving manipulating the platysma muscle flap with simple sutures to preserve the marginal mandibular nerve and superior cervical branches of the facial nerve while preventing fluid collection is described. After a submental incision and detachment of the platysma, 50–75% of the superficial lobe. The dissected space is then sealed by suturing the platysma muscle to the remaining gland and mylohyoid muscle, which helps minimize the risk of hematoma and sialoma. | Control group: 2 (8.00%)  Study group: 0 | Control group: 6 (24.00%)  Study group: 0 |  | 3 (1.25%) Paralysis of depressor of the lower lip  Control group: 1 (4.00%)  Study Group: 2 (0.93%): |  | 15 (6.25%)  Control Group: 15 (60.00%)  Study Group: 0 | Hemostatic sutures (transfixing and running) for preventing hematomas and seromas  No drains used |
| **Auersvald** | **9** | **2017** | **Retrospective** | Participants: 307 with SMG resection, 504 necklifts  Female: 461 (91.5%)  Male: 43 (8.5%)  Age: 54.3 (32-81)  Approach: Submental  Incision: Submental, 2-3 cm behind crease of chin  Monopolar: |  | 9 (2.93%)  Less than 20mL: 6 (1.95%)  Over 20 mL: 3 (1.95%) | 2 (0.65%): Resolved with needle aspirations |  |  |  |  | The continuous sutures placed around the superficial musculoaponeurotic system resection and closure areas explain there being no venous or arterial hematomas. |
| **Auersvald** | **10** | **2018** | **Retrospective** | Participants: 523 with SMG resection, 711 necklifts  Female: NR  Male: NR  Age: NR  Approach: Submental  Incision: Below to submental crease, 5.5 cm to most lateral part of SMG, 7cm to the submental crease  Monopolar | The fascia in the infrahyoid region is plicated at the hyoid and inferiorly, repositioning it superiorly and posteriorly to improve neck contour. The plication brings the superficial musculoaponeurotic system closer to the midline, facilitating access for resection. The gland is visualized, and the capsule is opened using blunt dissection with Metzenbaum scissors. After detaching the gland, it is mobilized using fine-tip electrocautery. Blood vessels encountered during the procedure are progressively cauterized to minimize bleeding. Expansion of the inferior capsule is recommended if there is insufficient space to manipulate the gland. | 0 | 2 (0.38%) | 1(0.19%) | 29 (5.54%): Transient weakness of the lower lip depressor muscle | 0 |  | Botulinum toxin was used to resolve sialocele in some cases. Continuous hemostatic sutures and absorbable sutures were utilized to close the gland capsule and prevent leakage. Nerve injury, which usually resolves within 90 days, may be alleviated with neurotoxin injection. |
| **O’Daniel** | **12** | **2021** | **Retrospective** | Participants: 152 with SMG resection out of 254 necklifts  Female: 225 (92%)  Male: 20 (8%)  Age: 52 (17-83)  Approach: Submental  Incision: Submental, At least 1.5cm behind the submental crease  Ligaclip | To prevent sialocele formation, a preventive technique was employed where 8 units of botulinum toxin were injected into the remaining gland, followed by reconstruction of the anterior capsule of the gland. |  | 3 (1.97%) |  |  |  |  | When the intraoperative use of *Botox* was temporarily discontinued, postoperative sialoceles occurred |
| **Talei** | **11** | **2024** | **Retrospective** | Participants: 72 with SMG resection out of 79 necklifts.  Female: 68 (94%)  Male: 4 (6%)  Age: 32-72  Approach: Submental  Incision: Submental, Behind the submental crease  Bipolar | To perform gland reduction, the medial and inferior portion is separated from the capsule, injected with local anesthesia, pulled medially, and partially resected using electrocautery. Irrigation is used intermittently to prevent heat damage to surrounding nerves. Reduction continues until the gland's bottom is level with the mylohyoid, above the hyoid, or deep to the mandible. Bleeding risk increases with more lateral and cephalic dissection due to larger vessels. | 3 (4.17%) |  |  |  |  |  | Hematomas treated with drainage and netting sutures.  There were no cases of sialomas or hematomas in the 260 cases of the author’s primary cases. There was also no use of neurotoxin, drain or gland imbrication. |
| **Su-Genyk** | **1** | **2024** | **Retrospective** | Participants: NR  Female: NR  Male: NR  Age: NR  Approach: Submental  Incision: Submental, Within submental crease or 1.5cm posterior  Monopolar: |  |  | 2.00-3.00% |  |  |  |  |  |
| **Basaran** | **5** | **2025** | **Retrospective** | Participants: 83 with SMG resection out of 294 facelifts  Female: 74  Male: 9  Age: 54  Approach: Submental  Incision: Over the submental crease  Bipolar | The study utilized the *LigaSure* bipolar energy-based instrument for submandibular gland excision. *LigaSure* was selected for its ability to seal vessels and connective tissue with minimal thermal spread (<2 mm), reducing the risk of damage to surrounding tissues. The instrument's efficacy in shortening operative times and minimizing blood loss was noted, particularly compared to monopolar and bipolar cautery, which had previously resulted in issues like charring, glandular inflammation, and extended surgical times. Excision was performed intracapsularly with avoidance of the posterior facial neurovascular bundle, using an average of 2–6 passes depending on the gland’s position. Post-excision, hemostasis was reinforced with *Surgicel* and drains were placed in both the subplatysmal and supraplatysmal spaces. | 2 (2.41%)  Resolved with aspiration | 0 | 0 | 7 (8.43%): Temporary lower lip weakness  6 (85.71%): Resolved in 3 months  1(14.29%): Persisted for up to 6 months |  | 6 (7.23%) | No Perioperative Glandular Bleeding  Improved Aesthetics: Reduced complications compared to conventional methods.  *LigaSure* minimized thermal injury compared to monopolar and bipolar cautery.  Improved Postoperative Outcomes: Attributed to *LigaSure*'s precise energy delivery and reduced glandular inflammation.  Patient Recovery: Temporary complications like nerve weakness were reversible with no permanent injuries reported. |
